# The significance of human respiratory syncytial virus (HRSV) in children from Ghana with acute lower respiratory tract infection: A molecular epidemiological analysis, 2006 and 2013-2014

**DOI:** 10.1371/journal.pone.0203788

**Published:** 2018-09-10

**Authors:** Evangeline Obodai, John Kofi Odoom, Theophilus Adiku, Bamenla Goka, Thorsten Wolff, Barbara Biere, Brunhilde Schweiger, Janine Reiche

**Affiliations:** 1 Department of Virology, Noguchi Memorial Institute for Medical Research, University of Ghana, Legon, Accra, Ghana; 2 Department of Infectious Diseases, Unit 17, Influenza and Other Respiratory Viruses, Robert Koch Institute, Berlin, Germany; 3 Department of Biomedical Sciences, School of Basic and Biomedical Sciences, University of Health and Allied Sciences, Ho, Volta Region, Ghana; 4 Department of Child Health, School of Medicine and Dentistry, College of Health Sciences, University of Ghana, Accra, Ghana; Kliniken der Stadt Köln gGmbH, GERMANY

## Abstract

**Background:**

Acute lower respiratory tract infection (ALRI) is a leading cause of childhood morbidity and mortality in developing countries. Globally, human respiratory syncytial virus (HRSV) is the most common pathogen of ALRI in infants and children. However, age-stratified HRSV disease burden data are largely absent from Africa, which is a key gap in informing an evidence-based recommendation for the introduction of an HRSV vaccine by the WHO.

**Methods:**

This study investigated the presence of HRSV in respiratory specimens from 552 children <5 years old with ALRI from Accra, Ghana in 2006 and 2013–2014 by real-time PCR. Of HRSV-positive samples the second hypervariable region of the viral G protein gene was sequenced and analyzed for phylogeny, characteristic amino acid substitutions, and potential glycosylation patterns. Further, HRSV infections have been characterized by age, symptoms and timely occurrence.

**Results:**

HRSV was observed in 23% (127/552) of the children with ALRI, with the highest incidence in infants younger than one year (33%, 97/295, *p* = 0.013). Within the observed seasonal circulation time of HRSV from June (mid-wet season) to December (beginning of the dry season) the incidence of ALRI due to HRSV was as high as 46% (125/273). HRSV disease was significantly associated with (broncho-) pneumonia, bronchiolitis, LRTI, and difficulty in breathing. Phylogenetic characterization of HRSV strains from Ghana identified the circulation of the currently worldwide prevailing genotypes ON1 and BA9, and shows evidence of an independent molecular evolution of ON1 and BA9 strains in Ghana resulting in potentially new subgenotypes within ON1 and BA9, provisionally named ON1.5, ON1.6, and BA9-IV.

**Conclusion:**

This study addresses important knowledge gaps in the forefront of introducing the HRSV vaccine by providing information on the molecular evolution and incidence of HRSV in Accra (Ghana, Africa).

## Introduction

Human respiratory syncytial virus (HRSV) is a member of the family *Pneumoviridae* and genus *Orthopneumovirus* [1]. The virus is enveloped, with a single-stranded, negative-sense RNA genome which contains 10 genes encoding 11 proteins. One of the proteins, the attachment glycoprotein G, is the most variable of the HRSV proteins, bearing two hypervariable regions (VR1 and VR2) [2]. The C-terminal end of the VR2 accounts for strain-specific epitopes and is therefore used for the description of molecular and evolutionary pattern [3]. Based on the reactivity of monoclonal antibodies against the G glycoproteins, HRSV has been classified into antigenic groups A and B (HRSV-A and HRSV-B) [4]. Genetic analysis of the G glycoprotein has further classified the two HRSV groups into several genotypes [2, 5, 6]. Currently known HRSV-A genotypes include GA1-GA7 [7, 8], SAA1 [9], SAA2 [10], NA1-NA4 [3, 11], CB-A [12], and ON1 [13]. The latest identified genotype ON1 (from Canada, 2010) contains a 72 nucleotide duplication in the VR2 region of the G glycoprotein [13]. Similarly, for HRSV-B, several genotypes have been described: GB1-4 [7, 8], GB5-GB13 [14, 15], SAB1-4 [9, 16], URU1 and URU2 [17], THB [18], CB-B [12], JAB1 [19] and BA [20, 21]. Genotype BA is characterized by a 60 nucleotide duplication within the VR2 region of the G protein gene, and was first identified in Buenos Aires (BA), Argentina in 1999 [20]. Since its discovery, BA strains have been shown to dominate and replace previously identified group B genotypes worldwide [10, 19, 22–28]. Evolution of genotype BA has so far resulted into several different clusters including BA1-6 [27], BA7-10 [29], BA11 [12], BA12 [23], BA13 [30], CB-1 [11], and BA-C [11].

HRSV is seasonally circulating in late fall or winter in temperate climates and generally during the rainy season in tropical and subtropical regions [12, 31–34]. Co-circulation of HRSV-A and–B has been observed in every season, with mostly group A predominating [7, 8, 28, 32]. Moreover, for each HRSV group, dominating genotypes were observed with shifting every few years [35].

HRSV has long been recognized as the chief causative agent of acute lower respiratory tract infection (ALRI) in children [35, 36]. Globally, 33.1 million episodes of ALRI were due to HRSV infection which resulted in estimated 3.2 million hospital admissions, and 59 600 in-hospital deaths in children younger than 5 years in 2015 [37]. Global studies suggested that the burden of incidence and severe disease for pneumonia is highest in Southeast Asia and Africa [38]. In Ghana, 13% of childhood deaths were due to acute respiratory infections [39, 40]. However, documentation of ALRI was based on clinical observations only, with no identification of the etiological agents involved. Indeed in Ghana, sentinel surveillance is restricted to influenza [41, 42] and investigations on the impact of respiratory viruses including HRSV are limited to few studies on ALRI [43–46]. WHO highlights that an HRSV vaccine is likely to be available in the next 5 to 10 years [37], however, WHO’s Strategic Advisory Group of Experts on Immunization have identified absence of age-stratified disease burden estimates and data for HRSV mortality in community from Africa and south Asia as the key gaps in informing an evidence-based recommendation for the introduction of an HRSV vaccine [37].

More importantly, this study determined incidences and clinical features of HRSV in hospitalized children with ALRI with respect to age, timely circulation of HRSV, and genetically characterized the circulating HRSV strains for the first time. These findings may contribute both to assess HRSV diagnostics and/or surveillance in affected age groups in the future and to the molecular understanding of the HRSV circulation in Ghana, Africa.

## Methods

### Study site

**Fig 1:**
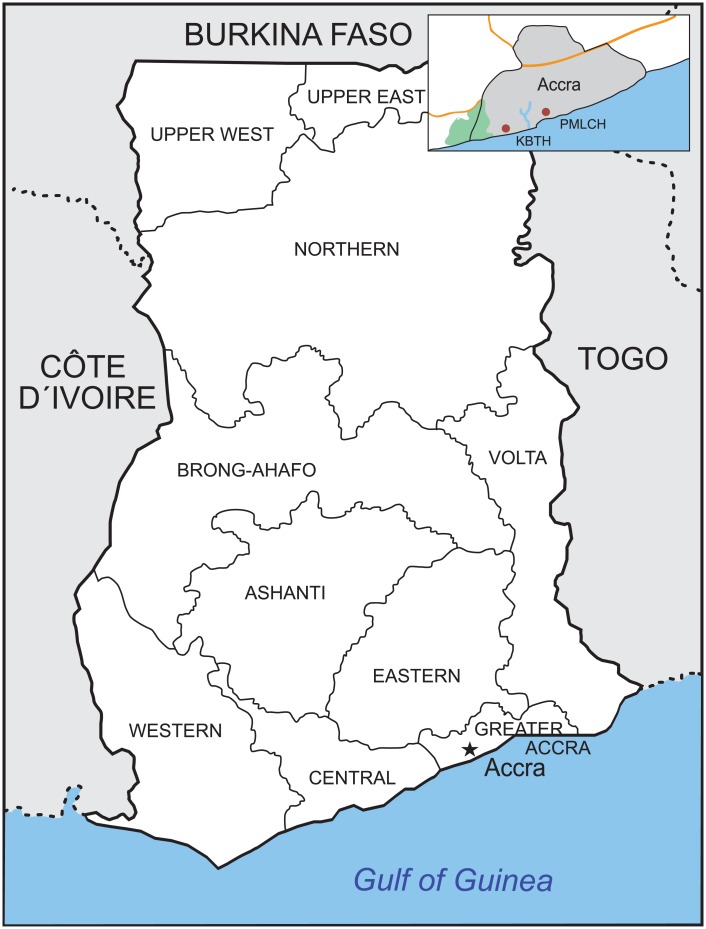
Ghana and its ten administrative regions. The two study sites, the Korle Bu Teaching Hospital (KBTH) and the Princess Marie Louise Children’s Hospital (PMLCH), were located in Accra within the Greater Accra region. Accra is mapped with a black star.

The study was conducted at two hospitals sited in Accra, Ghana, namely, the Korle Bu Teaching Hospital (KBTH), a tertiary and the leading national referral Centre of Ghana [47], and the Princess Marie Louise Children’s Hospital (PMLCH), a primary healthcare institution [48] (Fig 1).

### Study design and case definition

From February to November 2006 and January 2013 to December 2014, children younger than five years old with ALRI were prospectively enrolled. ALRI was defined as cough or nasal discharge or fever ≥ 38°C with fast breathing. The threshold for fast breathing was 60 breaths/minute (bpm) in children < 2 months old, 50 bpm in those 2–11 months, and 40 bpm in those 1–5 years [49, 50]. Known asthmatics and children with abnormal cardiovascular systems were excluded from this study.

Pediatricians and nurses collected relevant demographic and clinical information on admission.

### Specimen collection

From patients who met the ALRI case definitions nasopharyngeal aspirates or nasal swabs were collected. All specimens were immediately placed on ice and transported to the Department of Microbiology, University of Ghana Medical School and were stored at -80°C until dispatched to the National Influenza Centre at the Robert Koch Institute in Berlin, Germany, for virological and molecular investigations.

### Detection of HRSV

Upon arrival in the laboratory, 3ml of sterile minimal essential medium containing 100U/ml penicillin-streptomycin was added to each sample and vortexed. Samples were aliquoted and stored at -70°C until further use. RNA was extracted from 400μl of diluted specimen using RTP DNA/RNA Virus Mini Kit (Invitek, Germany). Reverse transcription was performed with 25μl of RNA using 200U M-MLV Reverse Transcriptase (Invitrogen, Germany) in a total reaction volume of 40μl as described previously [32]. Triplex real-time PCR assay was performed to detect HRSV-A and–B, influenza A virus (IAV), and the internal extraction control with qPCR detection limits of 9 genome equivalents per reaction for HRSV and 4 for IAV. For HRSV, the N gene was amplified. PCR was performed in a 25μl reaction mixture containing 3μl of cDNA, 300nM of HRSV-1084 (forward primer, 5’GATGGCTCTTAGCAAAGTCAAGTT3’) and 1200nM HRSV-1253 (reverse primer, 5’CATCTTCWGTGATTAATARCATRCCACATA3’), 150nM HRSV–MGB (probe, VIC-ACAGGAGATARTATTGAYACTC, Applied Biosystems, USA), 300nM of M+25, 100nM of M-124sw, 1200nM M-124BB, 100nM M+64 MBG (probe, FAM), 300nM of FCV F54 and FCV R141, and 100nM FCV TM96 (probe, LC610), 200μM deoxynucleoside triphosphates with dNTP, 5mM MgCl_2_, 0.5U of Platinum *Taq* DNA polymerase (Invitrogen, Germany), and 1x PCR buffer [51]. Amplification was carried out at 95°C for 5 min, and 45 cycles of 15 sec at 95°C and 30 sec at 60°C. Afterwards, HRSV-positive samples were differentiated into HRSV-A and -B by group specific real-time multiplex RT-PCR targeting the G gene as described previously [32]. In short, 3μl of cDNA was amplified with 600nM HRSVA-G409 (forward primer, 5’AAGACCAAAAACACAACAACAA3’), 300nM HRSVA-G586 (reverse primer, 5’TTGGTATTCTCTTGCAGATGG3’), HRSVA-G556 (probe, YAK-5’TTGGATTGTTGCTGCATATGCTGCT3’-BBQ), 300nM HRSVB-G155 (forward primer, 5’CAATGATAATCTCAACCTCTCTCA3’), 300nM HRSVB-G303 (reverse primer, 5’GGTGAGACTTGAGTAAGGTAAGTG3’), 150nM HRSVB–G201 (probe, 5’6FAM-CATCTCTGCCAATCACAAAGTTACACTAACAAC3’-BBQ), and 1U of Platinum *Taq* DNA polymerase (Invitrogen, Germany) in a 25μl reaction. Amplification conditions were as described above.

### Sequencing of HRSV

Differentiated HRSV-A and -B positive samples were selected for amplification of the second hypervariable region (VR2) of the G protein gene by either external or nested PCR [32]. HRSV-A external PCR primers were HRSVA-G513F (5’AGTGTTCAACTTTGTACCCTGC3’) and HRSVA-F131R (5’CTGCACTGCATGTTGATTGAT3’), and nested PCR primers were HRSVA-G606F (5’AACCACCACCAAGCCCACAA3’) and HRSV-F22R (5’CAACTCCATTGTTATTTGCC3’).

HRSV-B external PCR primers were HRSVB-G524F (5’TTGTTCCCTGTAGTATATGTG3’) and HRSV-F55R (5’AGTTAGGAAGATTGCACTTGA3’), and nested PCR primers were HRSVB-G603F (5’AAAACCAACCATCAAACCCAC3’) and HRSV-F22R. The PCR products were purified with MSB^®^Spin PCRapace purification kit (Stratec Molecular GmbH, Germany) and PCR amplicons which were excised from agarose gel were purified with Invisorb^®^ Spin DNA Extraction kit (Stratec Molecular GmbH, Germany) according to the manufacturer’s instructions. Purified PCR products were cycle sequenced in the forward and reverse directions with primers of the external or nested PCR using the BigDye Terminator v3.1 cycle sequencing kit (Applied Biosystems).

### Sequence analyses

A multiple sequence alignment was compiled from the VR2 region of the G protein gene of HRSV-A (nt 634 –nt 969, reference strain AUS/A2/61 (Genbank accession number: M11486) and -B (nt 652 –nt 975, reference strain 18537 (Genbank accession number: M17213) using ClustalW in MEGA version 5.2. Maximum-likelihood trees were calculated with best-fit substitution model Tamura-Nei (TN93+G) for HRSV-A, and Hasegawa-Kishino-Yano (HKY+G) model for HRSV–B, respectively. The reliability of the branching order was estimated by performing 1000 bootstrap replicates. The trees were manually edited in Microsoft PowerPoint2010. Deduced amino acid sequences were translated with the standard genetic code using Bioedit version 7.2.5. To estimate the numbers of potentially N- and O-glycosylated serine and threonine residues, the NetNGlyc 1.0 (http://www.cbs.dtu.dk/services/NetNGlyc/) and NetOGlyc 4.0 (http://www.cbs.dtu.dk/services/NetOGlyc/) servers were used, respectively [52]. Synonymous and nonsynonymous mutations were analyzed by the method of Nei and Gojobori [53].

### Nucleotide sequence accession numbers

The GenBank accession numbers of HRSV nucleotide sequences obtained in the present study are KY910974 to KY911080.

### Statistical analyses

The impact of age, gender, and type of admission on the frequency of HRSV infections as well as the correlation between clinical diagnosis and clinical symptoms with HRSV infection was analyzed using the chi-squared test. The statistics were performed with the IBM SPSS version 22 and STATA version 13 softwares. Statistical significance was assessed by the *p*-value analysis. *P*-values < 0.05 were considered to be statistically significant.

### Ethical consideration

The study protocol was approved by the Ethical and Protocol Review Committee of the University of Ghana Medical School, College of Health Science (MS-Et/M.7.1—P.4/2006-07; MS-Et/M.7 –P 4.10/2012-2013). The parents or caregivers provided written informed consent for their children to participate in this study.

## Results

### Study group

From February to November 2006 and January 2013 to December 2014, 552 children met the ALRI inclusion criteria. Patients were primarily residents of the Greater Accra Region (88%), followed by residents of the Central (8%) or Eastern Region (1.2%). Patients from Volta, Ashanti, Western or Brong-Ahafo regions were rarely enrolled (<1%) (Fig 1).

Children were divided into five age groups (0<1: 295, 1<2: 132, 2<3: 76, 3<4: 31, 4<5: 18; most patients (53%) enrolled were children younger than one year.

### ALRI and HRSV case distribution

**Fig 2:**
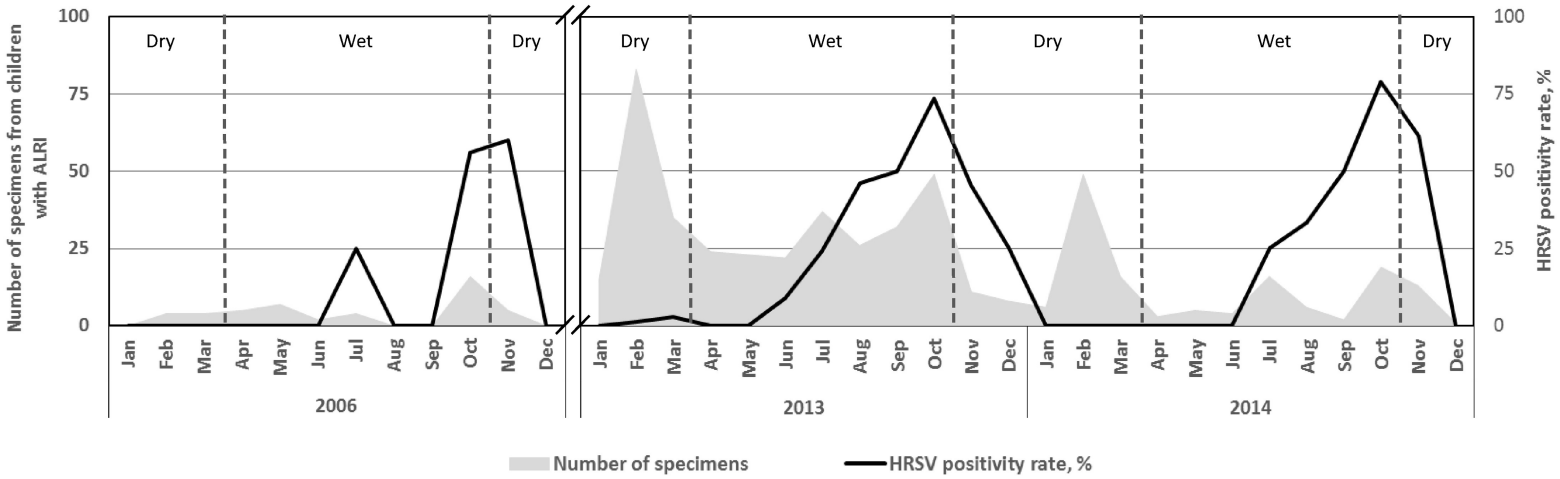
Monthly distribution of specimens from children with ALRI and HRSV positivity rate. Dry and wet seasons are indicated.

Specimen collection occurred all through the year with highest collection numbers in February, July, and October (Fig 2). HRSV activity was primarily from June/July to November/December with peak activities of 60% to 79% in October/November of each year.

The climate of Ghana is tropical and generally characterized by a wet season from April to October and a dry hot season from November to March (Fig 2). This suggests that HRSV both circulates from the mid-wet season to the early dry season in Ghana and is a causative agent of ALRI in children in the second half of the year, mainly in October.

### Characteristics of HRSV-positive patients

Within the period of investigation, 23% (127/552) of the children with ALRI were positive for HRSV. Of the 127 children, 88% were inpatients (*p* = 0.007) (Table 1). Boys constituted 58% and the mean age in this study was 0.92 years (range 0–5 years). The highest number of positive individuals was amongst infants younger than 1 year (33%, 97/295, *p* = 0.013), followed by children aged between 1 and 2 years (12%, 16/132) and between 2 and 3 years (17%, 13/76), respectively. As shown in Table 1, the incidence of HRSV decreased with increasing age; HRSV was detected in only one child in the age of 3 to 5 years.

**Table 1:** Demographic and clinical characteristics of HRSV positive patients, 2006 and 2013–2014.

Patients‘ characteristics	N (%)	*p*-value
**Age in years**^a^		
0<1	97 (33)	**0.013**
1<2	16 (12)	0.628
2<3	13 (17)	0.853
3<4	0	0
4<5	1 (6)	0.381
**Gender**		
Boys	73 (57)	0.486
Girls	54 (43)	
**Department**		
Outpatients	15 (12)	**0.007**
Inpatients	112 (88)	
**Clinical diagnosis**		
Bronchopneumonia	101 (80)	**<0.01**
Pneumonia	12 (9)	**0.023**
Bronchiolitis	19 (15)	**0.019**
LRTI^b^, unclassified	18 (14)	**0.002**
Respiratory distress	7 (6)	0.667
Difficulty in breathing^c^	71 (56)	**0.002**

^a^ Within each age group the prevalence was calculated as the proportion of the number of HRSV positive specimens out of the number of investigated specimens per age group.

^b^ LRTI, lower respiratory tract infection

^c^ Difficulty in breathing was defined as chest indrawing.

HRSV-positive children were significantly associated with bronchopneumonia, pneumonia, bronchiolitis, and lower respiratory tract infection (LRTI) (Table 1). Moreover, HRSV infection in children was significantly associated with difficulty in breathing.

### Predominant HRSV group and genotypes

HRSV was detected in 21%–28% of the specimens within each study year (Table 2). HRSV-B predominance was determined in 2006 (85%) and 2013 (80%), whereas HRSV-A was solely detected in 2014.

**Table 2:** Distribution of HRSV groups and genotypes, by year.

Year	N of specimens	N (%) of HRSV positive specimens	N (%) of HRSV group A viruses	N (%) of HRSV group B viruses	HRSV-A genotypes, N			HRSV-B genotypes, N	
					ON1	NA1	SAA2	BA9	SAB4
**2006**	47	13 (28)	2 (15)	11 (85)	-	-	1	5	-
**2013**	365	84 (23)	17 (20)	67 (80)	13	4	-	55	1
**2014**	140	30 (21)	30 (100)	0	27	1	-	-	-
**Total**	552	127 (23)	49 (39)	78 (61)	40	5	1	60	1

**Fig 3:**
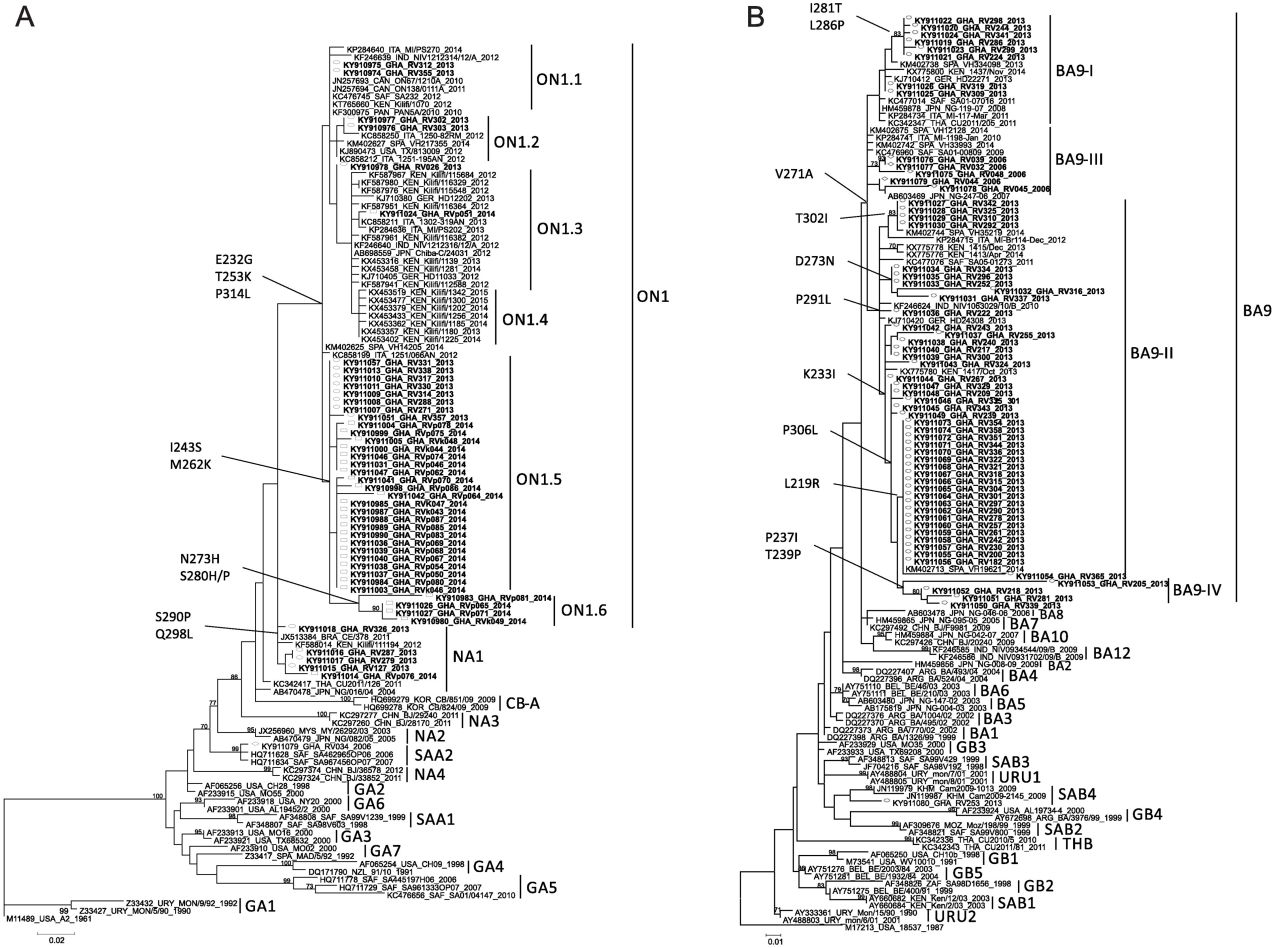
Maximum likelihood analysis of the VR2 region of HRSV G protein gene sequences from Ghana. The phylogenetic trees were constructed with MEGA version 5.2 using the Tamura-Nei and the Hasegawa-Kishino-Yano methods for (a) HRSV-A and (b) HRSV–B, respectively, with 1,000 replicates. Reference sequences representing the different HRSV genotypes were obtained from GenBank and are indicated by their accession numbers. Sequences from this study are shown in bold and designated by the geographic location (GHA), patient number and year of collection. The genotype clusters are indicated on the right of each figure. Only bootstrap values greater than 70% are displayed at the branch nodes. Characteristic amino acid substitutions are indicated at branch nodes.

For phylogenetic analyses, sequencing of the VR2 region of the glycoprotein G gene was performed on 46 HRSV-A and 61 HRSV-B viruses (Fig 3). HRSV-A virus strains clustered into three genotypes: ON1 (n = 40), NA1 (n = 5), and SAA2 (n = 1). SAA2 was last recorded in 2006, whereas ON1 and NA1 co-circulated in 2013 and 2014, with ON1 predominating in each of these years (Table 2). Phylogenetic analyses showed that all HRSV-B virus strains belonged to genotype BA9; except for one virus (GHA/RV253/2013) from the season 2013 which clustered within genotype SAB4 (Fig 3).

### Molecular characterization

**Fig 4:**
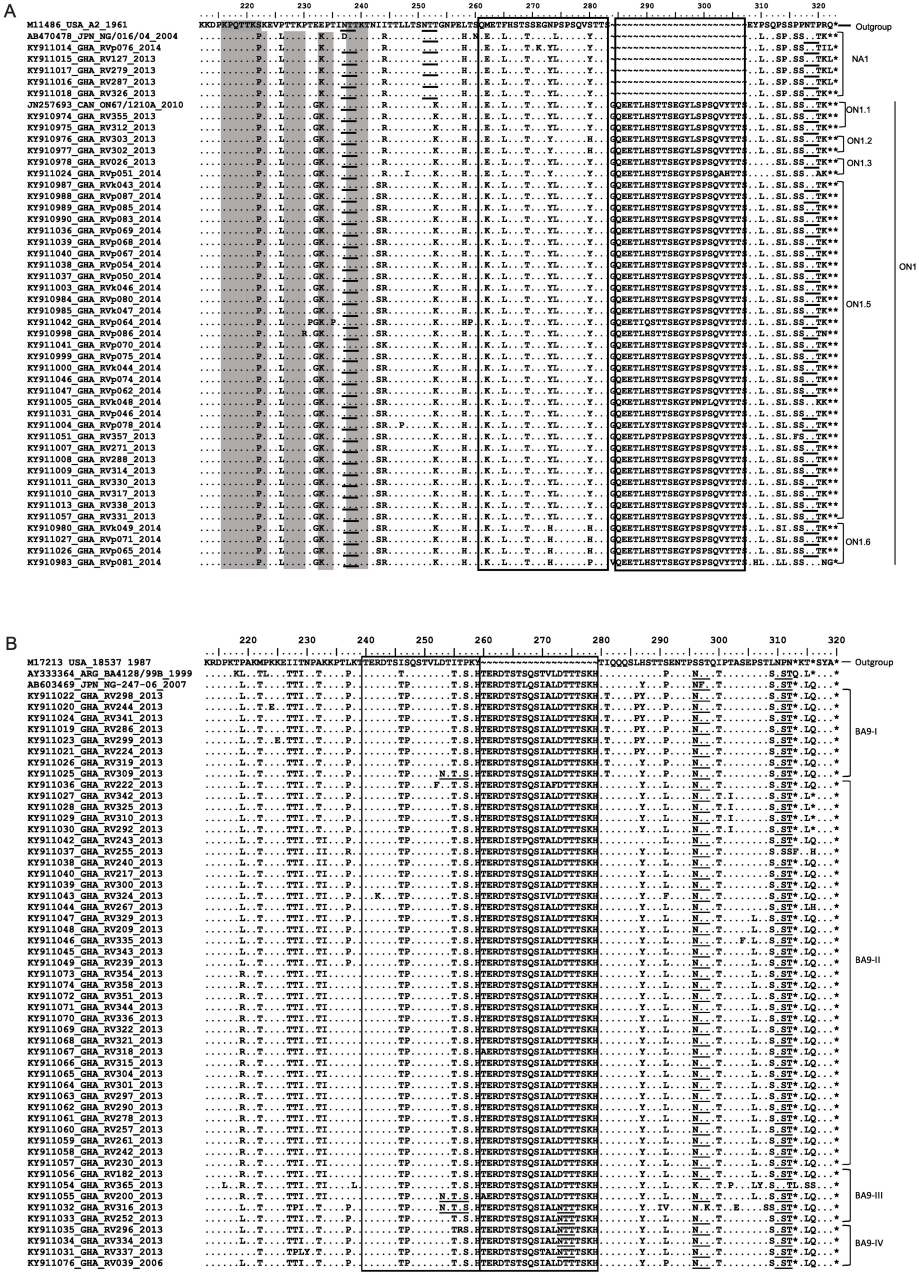
Deduced amino acid alignment of the VR2 region of HRSV G protein gene sequences. Alignments of (a) HRSV- group A and (b) HRSV group B viruses from Ghana are shown in relation to prototype strains, and genotype specific strains. Identical residues are indicated by dots. Stop codons are indicated by asterisks. Rectangles indicate the two copies of amino acid duplicated regions. Potential N-glycosylation sites (NXT/S, where X is not a proline) are underlined. Potential sites for extensive O-glycosylation KPXnTTKXn motifs (where X is any amino acid) are indicated by gray shading.

Of the VR2 region of the G protein gene, the deduced amino acid sequence of HRSV genotypes A (NA1 and ON1) and genotype B (BA9) from Ghana were compared with A2, NG-016-04 (NA1), ON67-1210A (ON1) (Fig 4a) and 18537, BA4128/99B (BA), and NG-247-06 (BA9) (Fig 4b) reference strains, respectively. In comparison to NA1 viruses from Niigata, Japan [3], HRSV from Ghana displayed a different stop codon at position 323 (299 without duplication), which is due to a single nucleotide mutation at position 322 resulting in amino acid leucine instead of a stop codon. Genotype ON1 is characterized by a 72 nt duplication in the C-terminal end of the G gene, resulting in 24 extra amino acids (GQEETLHSTTSEGYLSPSQVYTTS) spanning positions 284–307 compared to prototype strain A2 [13] (Fig 4a). All ON1 viruses from Ghana displayed an early stop codon at position 322 as initially described [13], except for one virus strain (GHA/RVp081/2014, Fig 4a). Originally, three genotype specific ON1 substitutions have been identified (E232G, T253K, P314L (P290L without duplication) [13], all of them could be displayed in ON1 viruses from Ghana (Fig 4a). For some ON1 virus strains from Ghana, one (S280H), two (I243S, M262K) or four (I243S, M262K, N273H, S280H/P) additional substitutions have been observed (Fig 4a), when compared to HRSV-A2 strain and ON1 strain, respectively. Interestingly, ON1 viruses from Ghana characterized by the additional two and four substitutions, respectively, clustered into two potential new subgenotypes within ON1, here provisionally denoted as ON1.5 and ON1.6 (Fig 3). The genetic divergence (*p* distance) within ON1.5 and ON1.6 was 0.008 and 0.021, respectively, and ranged between 0.031 and 0.04 as well as between 0.014 and 0.031 to the other ON1 subgenotypes. Genetic distances were similar to previously defined ON1 subgenotypes.

Genotype BA9 viruses of HRSV-B are evolutionary descendants of the original BA genotype, which contain a 60 nt duplication in the VR2 region of the G gene, resulting in 20 additional amino acids (TERDTSTSQSTVLDTTTSKH) spanning aa 260 –aa 279 [20, 21] (Fig 4b). Just as for original BA strains [21], BA9 viruses from Ghana have a predicted protein length of 313 aa, although few BA9 strains use alternative stop codons at position 320. For all BA9 viruses from Ghana the genotype BA9 specific substitution V271A was identified. In comparison to the BA4128/99B original strain, some BA9 viruses from Ghana showed additional substitutions observed elsewhere, e.g. L219R, K233I, I281T, L286P, P291L, T302I, P306L [26, 29, 54, 55]. Moreover, few substitutions were identified being apparently unique to BA9 viruses from Ghana (P237I, T239P, D273N, and T302I) leading to separate clusters. Calculation of the genetic divergences identified a potential new BA9 subgenotype, here provisionally denoted as BA9-IV (Fig 3). BA9-IV is characterized by the additional amino acid substitutions P237I and T239P and its genetic divergence (*p* distance) of 0.018 within BA9-IV and its range between 0.034 and 0.045 to the other BA9 subgenotypes.

Moreover, different pattern of putative N- and O-glycosylation sites were predicted among HRSV from Ghana. Three N-glycosylation sites (NXT, where X is not proline) were identified among NA1 viruses (aa positions 237, 251, and 318), whereas two of these N-glycosylation sites (aa positions 237 and 318) were also identified in most ON1 viruses (Fig 4a). For HRSV BA9 strains, generally two sites were identified at aa 296 and aa 310, which might obviously be conserved among other HRSV BA strains [3]. Additional N-glycosylation sites were identified in few BA9 strains at aa 253 and aa 273. The analysis for potential attachment sites for O-linked sugars predicted 32 residues in NA1 and 37 to 41 residues in ON1 strains, respectively. Within BA9 strains 40 to 45 predicted sites were identified. In addition to serine and threonine residues, three repeats of the motif KPX_n_TTKX_n_ (where X is any amino acid) (Fig 4) were present among NA1 and ON1 viruses and may be associated with extensive O-glycosylation of the G protein [56].

## Discussion

ALRI is a leading cause of childhood morbidity and mortality in developing countries including Ghana [57]. Disease burden data in Ghana are largely missing and etiological agents are rarely identified and/or are limited to few pathogens [41, 44, 45].

In this study, respiratory specimens from children younger than 5 years old with ALRI were prospectively collected in two study sites in Accra, Ghana. The highest number of specimens was collected in infants younger than one year old (53%, 295/552), reflecting that the burden of ALRI is highest in very young children. Similarly, estimates of incidence of hospital admissions for severe ALRI determined a 2.2 times higher admission rate in infants aged 0 to 11 months (50.8 episodes per 1000 per year) than the overall rate in young children aged 0 to 59 months (22.6 episodes per 1000 per year) in Africa [57]. The same observation was made for industrialized countries but with generally lower incidence rates [57]. Investigation of specimens of the children with ALRI from this study observed an average positivity rate of 23% (127/552). More interestingly, the highest number of positive individuals was amongst infants younger than one year, followed by children aged between one and three years. This implicates that both the incidence of HRSV is decreasing with increasing age [32, 37] and HRSV plays a major role in causing ALRI in children younger than three years old in the study population.

With respect to the seasonal circulation of HRSV from the mid-wet season to the beginning of the dry season (June to December), incidence of ALRI due to HRSV in these months was as high as 46% (125/273). This highlights that, at that time of the year, HRSV is indeed an important causative agent of ALRI in the Greater Accra Region, Ghana. A similar circulation pattern was observed for other countries from sub-Saharan Africa, e.g. Senegal and Cameroon [33, 58]. In countries from different tropical regions, HRSV circulation is also associated with increased rainfall [59, 60]. Nonetheless, there were a few studies from the tropics which demonstrated a HRSV correlation with the dry season [61, 62]. In Africa, data on HRSV circulation are limited and the precise reasons for the strong seasonality remain to be determined. It might be speculated that during the rainy season, children tend to be kept indoors and that crowding may account for the increased incidence and activity of HRSV during this period. Further, high humidity might be conducive to viral survival by both preventing drying and loss of infectivity of the virus. HRSV is known to be a labile virus, which does not survive well under high temperatures; this might explain the relationship between wet and/or cooler weather [63].

HRSV-positive children from Ghana were significantly associated with (broncho-) pneumonia, bronchiolitis, and LRTI. Difficulty in breathing was also of significance in these children. HRSV-associated bronchiolitis and (broncho-) pneumonia were high among children with ALRI from both industrialized and developing countries, and were commonly referred to as typical HRSV symptoms [36, 57, 64].

Differentiation of circulating HRSV strains from Ghana revealed a predominance of group B over HRSV group A viruses in two (2006 and 2013) of the three studied years. Worldwide, a generally higher incidence for HRSV group A is described with at least two consecutive seasons of predominance of HRSV-A [10, 32]. In this study, only two consecutive seasons have been monitored, and therefore, a regular pattern of group dominance could not be observed.

In Ghana, HRSV-A strains clustered with genotype SAA2 in 2006 and with NA1 and ON1, respectively, in 2013 and 2014. With respect to the two consecutive seasons, the new genotype ON1 has continued to rise and obviously might have displaced its ancestor NA1 as reported elsewhere [26, 54]. Phylogenetic characterization of ON1 strains from Ghana revealed the development of two potential new subgenotypes within ON1, ON1(1.5) and ON1(1.6), in 2013 and 2014, which might explain the displacement of genotype NA1 in Ghana. The novel ON1 genotype was first detected in December 2010, in Ontario, Canada [13]. Studies from Italy [65], China [54], Senegal [55] and Kenya [66] report on the occurrence of ON1 for the years 2011, 2012 or 2013, suggesting that ON1 strains might have been introduced into Ghana before 2013. For the two years of HRSV group B detections in Ghana, almost all HRSV-B strains clustered within genotype BA9. Genotype BA9 is an ancestral descendant of the BA genotype and was first described in Niigata, Japan in the season 2006/07 [29]. Since then it rapidly spread around the world and became a dominant group B genotype in many countries [10, 24, 26, 67, 68]. Interestingly, no HRSV-B strains were detected in Ghana in 2014, in spite of 30 HRSV detected at that time. It appears that the rise of genotype ON1 not only displaced NA1 strains but also excluded group B strains. Similarly, the introduction of ON1 resulted in a replacement of HRSV-A genotype GA2 and, moreover, in a change of the HRSV subgroup alternation pattern (AAB → AAA) in Kilifi, Kenya, in the season 2012/13 [66]. However, reasons for this effect are so far unknown [66].

Based on the phylogenetic and deduced amino acid sequence analysis, accumulations of amino acid substitutions in HRSV ON1 strains from Ghana over two consecutive seasons have been observed. In 2013, two strains resemble the original ON1 strains with the characteristic substitutions E232G, T253K, and P314L. Other ON1 strains from 2013 acquired one (S280H) or two (I243S, M262K) additional substitutions. The latter two substitutions have also been detected for the bulk of ON1 strains from 2014. ON1 strains from 2013 and 2014 harboring these substitutions clustered in a potential new ON1 subcluster, ON1.5. Interestingly, there were four virus strains, which acquired two more additional substitutions (N273H, S280H) and build the second potential new ON1 subcluster, ON1.6. Position 280 has been previously identified as a positive selected evolving site [26], whereas amino acid substitution N273H has been observed for the first time. The evolution of the major antigens of HRSV might be associated with selective pressure due to host immune response [26]. It can be speculated that the accumulation of additional amino acid substitutions like N273H and S280H led to an enhanced ON1 circulation and to the drastic reduction of HRSV-B in Ghana in 2014.

However, for HRSV-BA9 strains from Ghana different amino acid substitutions have been detected; some of them were apparently new. Amino acid substitutions P237I and T239P were characteristic for the potentially new BA9-IV subgenotype represented by strains from Ghana. For the short period of molecular HRSV investigation in Ghana, it is astounding how many amino acid substitutions have been observed for both HRSV genotype ON1 and BA9 strains. Search for phylogenetic signatures of selective pressure in HRSV-A and–B showed that HRSV genes have predominantly negatively or neutrally selected evolving sites and only a minority was under positive selection [26]. HRSV variants are quickly evolving and spreading around the world, however, additional investigations are needed to evaluate whether amino acid changes in the G protein may help the virus to alter the antigenic characteristics leading to an evolutionary advantage [26].

HRSV glycosylation is an important hallmark of antigenicity of the virus and is highly variable between HRSV strains [69]. Within ON1 strains, two potential N-glycosylation sites were conserved at amino acid positions 237 and 318 [13]. For two ON1 strains from Ghana the second site has been lost due to a substitution at aa 320. Originally, two potential N-glycosylation sites were described for BA9 viruses [29]. Few BA9 strains were detected to have either an additional N-glycosylation site (D253N, D273N) or lost one site (N296K/Y, T298K/A) (Fig 4). Changes for amino acid positions 296 and 298 have also been detected in HRSV strains from Cambodia, China and Italy [15, 16, 26]. Their influence on antibody recognition and immune response is still to be elucidated.

## Conclusions

This study observed the highest burden of ALRI in infants and determined the highest incidence of HRSV in children aged between zero and three years in the Greater Accra region in Ghana. The identification of the seasonal circulation of HRSV and the association of clinical diagnoses with HRSV infection enable to set up targeted diagnostics in these age groups, e.g. with rapid tests. Moreover, this first phylogenetic characterization of HRSV strains from Ghana identified the circulation of the worldwide prevailing genotypes ON1 and BA9, respectively, for the years 2006 and 2013–2014, and shows evidence for an independent molecular evolution of HRSV group A and B strains in Ghana. These findings will not only improve the understanding of the molecular evolution of HRSV especially in sub-Saharan Africa, but moreover provide evidence for the burden of HRSV in this region of the world, which is necessary for rational recommendations leading to the introduction of the HRSV vaccine. However, implementation of a standardized surveillance will be necessary to achieve this goal.
