# Correction: The significance of human respiratory syncytial virus (HRSV) in children from Ghana with acute lower respiratory tract infection: A molecular epidemiological analysis, 2006 and 2013-2014

**DOI:** 10.1371/journal.pone.0221315

**Published:** 2019-08-14

**Authors:** Evangeline Obodai, John Kofi Odoom, Theophilus Adiku, Bamenla Goka, Thorsten Wolff, Barbara Biere, Brunhilde Schweiger, Janine Reiche

For Figs 3 and 4, several sequences were associated with the wrong accession number. Please see the correct Figs 3 and 4 below.

**Fig 3:**
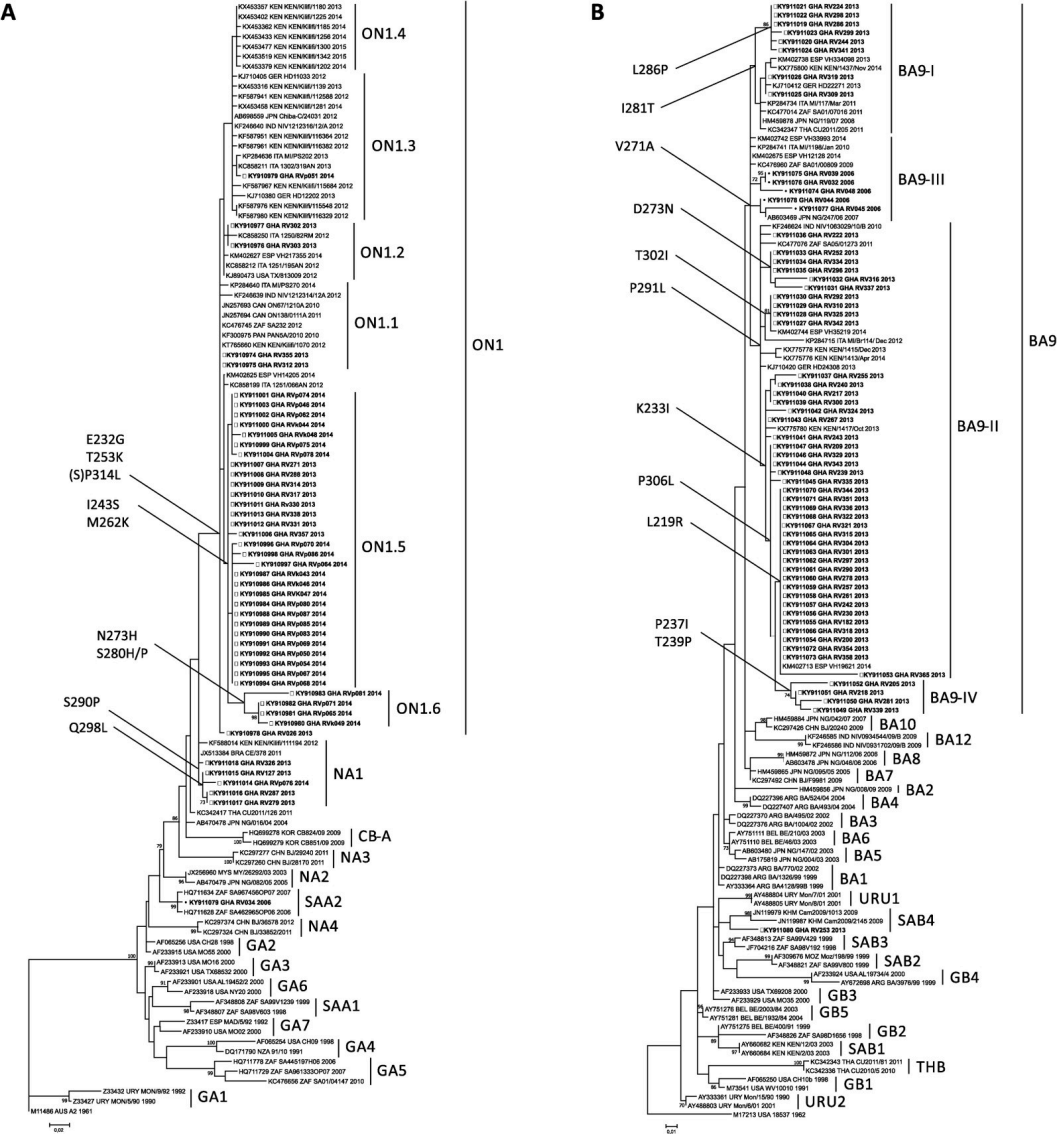
Maximum likelihood analysis of the VR2 region of HRSV G protein gene sequences from Ghana. The phylogenetic trees were constructed with MEGA version 5.2 using the Tamura-Nei and the Hasegawa-Kishino-Yano methods for (a) HRSV-A and (b) HRSV–B, respectively, with 1,000 replicates. Reference sequences representing the different HRSV genotypes were obtained from GenBank and are indicated by their accession numbers. Sequences from this study are shown in bold and designated by the geographic location (GHA), patient number and year of collection. The genotype clusters are indicated on the right of each figure. Only bootstrap values greater than 70% are displayed at the branch nodes. Characteristic amino acid substitutions are indicated at branch nodes.

**Fig 4:**
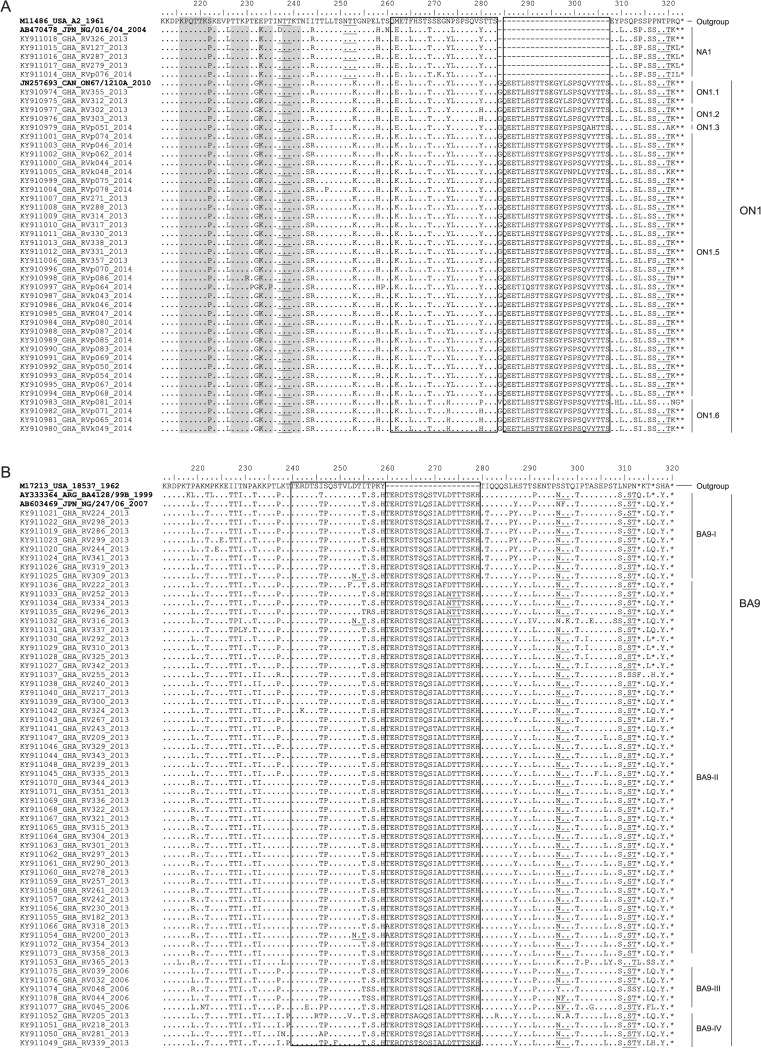
Deduced amino acid alignment of the VR2 region of HRSV G protein gene sequences. Alignments of (a) HRSV- group A and (b) HRSV group B viruses from Ghana are shown in relation to prototype strains, and genotype specific strains. Identical residues are indicated by dots. Stop codons are indicated by asterisks. Rectangles indicate the two copies of amino acid duplicated regions. Potential N-glycosylation sites (NXT/S, where X is not a proline) are underlined. Potential sites for extensive O-glycosylation KPXnTTKXn motifs (where X is any amino acid) are indicated by gray shading.
